# Rhodopsin-mediated nutrient uptake by cultivated photoheterotrophic *Verrucomicrobiota*

**DOI:** 10.1038/s41396-023-01412-1

**Published:** 2023-04-29

**Authors:** Rinat Bar-Shalom, Andrey Rozenberg, Matan Lahyani, Babak Hassanzadeh, Gobardhan Sahoo, Markus Haber, Ilia Burgsdorf, Xinyu Tang, Valeria Squatrito, Laura Gomez-Consarnau, Oded Béjà, Laura Steindler

**Affiliations:** 1 Department of Marine Biology, Leon H. Charney School of Marine Sciences, University of Haifa, Haifa, 3498838 Israel; 2 Faculty of Biology, Technion-Israel Institute of Technology, Haifa, 3200003 Israel; 3 Department of Biological Sciences, University of Southern California, Los Angeles, CA 90089 USA; 4 Centro de Investigación Científica y de Educación Superior de Ensenada, Ensenada, BC México; 5 Present Address: Department of Ecology and Environmental Sciences, School of Life Sciences, Pondicherry University, Puducherry, 605014 India; 6 Present Address: Institute of Hydrobiology, Biology Centre CAS, Na Sadkach 7, 37005 Ceske Budejovice, Czechia

**Keywords:** Water microbiology, Microbial biooceanography

## Abstract

Rhodopsin photosystems convert light energy into electrochemical gradients used by the cell to produce ATP, or for other energy-demanding processes. While these photosystems are widespread in the ocean and have been identified in diverse microbial taxonomic groups, their physiological role in vivo has only been studied in few marine bacterial strains. Recent metagenomic studies revealed the presence of rhodopsin genes in the understudied *Verrucomicrobiota* phylum, yet their distribution within different *Verrucomicrobiota* lineages, their diversity, and function remain unknown. In this study, we show that more than 7% of *Verrucomicrobiota* genomes (*n* = 2916) harbor rhodopsins of different types. Furthermore, we describe the first two cultivated rhodopsin-containing strains, one harboring a proteorhodopsin gene and the other a xanthorhodopsin gene, allowing us to characterize their physiology under laboratory-controlled conditions. The strains were isolated in a previous study from the Eastern Mediterranean Sea and read mapping of 16S rRNA gene amplicons showed the highest abundances of these strains at the deep chlorophyll maximum (source of their inoculum) in winter and spring, with a substantial decrease in summer. Genomic analysis of the isolates suggests that motility and degradation of organic material, both energy demanding functions, may be supported by rhodopsin phototrophy in *Verrucomicrobiota*. Under culture conditions, we show that rhodopsin phototrophy occurs under carbon starvation, with light-mediated energy generation supporting sugar transport into the cells. Overall, this study suggests that photoheterotrophic *Verrucomicrobiota* may occupy an ecological niche where energy harvested from light enables bacterial motility toward organic matter and supports nutrient uptake.

## Introduction

The classic view that most of the light energy capture for metabolism in the ocean is carried out by photosynthetic microorganisms and used for carbon fixation has been challenged by the discovery of proteorhodopsin photosystems (PR) in marine prokaryotes [1–3]. PRs are light-activated proton pumps that create a proton motive force (*pmf*) across the cellular membrane [1, 3]. Depending on the microorganism, the resulting *pmf* can be used for a variety of physiological functions, such as producing biochemical energy (ATP) to contribute to metabolism and growth [4], supporting cellular transport and other energy-demanding cellular processes (e.g., proton-mediated substrate uptake [5] and ATP-mediated substrate uptake, as in ABC transporters [6]), and enhancing cell survival during respiratory stress [7].

The importance of PR photosystems in marine bacteria is supported by a global analysis of marine metagenomes, which showed that for small free-living marine microorganisms (0.2–0.8 μm fraction) the proteorhodopsin coding gene (*pr*) exceeds by threefold the combined abundance of oxygenic and anoxygenic photosynthesis genes [8]. This high abundance of PRs among bacterioplankton suggests that a significant amount of solar radiation is harvested by these photoreceptive proteins. Supporting this notion, using the environmental concentration of the chromophore retinal as a proxy for PR concentration, a recent study showed that PRs potentially absorb as much light energy as chlorophyll-*a*-based phototrophy in the water column [9]. The *pr* genes are widespread in bacteria from virtually all oceanic regions [10**–**14] and PR-mediated photoheterotrophy may be the dominant prokaryotic metabolism in the photic zone [13], particularly in ultraoligotrophic regions such as the Eastern Mediterranean Sea [15].

Among microbial rhodopsins, PRs are the most abundant and widespread type in marine systems, being present in a wide range of prokaryotic taxa. Of the other subfamilies of proton pumps that constitute a smaller fraction of rhodopsins in the sea, the most prevalent is the subfamily of xanthorhodopsins (XRs) [16]. The simplicity of the rhodopsin proton pump gene system, which only requires six genes [3], contributes to the ease with which they are horizontally transferred across diverse taxonomic groups [17], and consequently to its pervasiveness in the marine ecosystem. Accordingly, PRs are found in diverse taxonomic groups characterized by distinct metabolic capacities that allow them to occupy separate ecological niches within the microbial community. Marine PR-containing prokaryotes have been found among *Actinomycetota*, *Bacteroidota*, and *Alpha*-, *Beta*- and *Gammaproteobacteria*, as well as marine *Euryarchaeota*. However, most microorganisms within these taxa are not yet available in culture, which has limited the ability to study how rhodopsin-mediated light harvesting modulates microbial physiology and ecology.

So far, the influence of light on PR phototrophs has only been studied in a small number of marine strains, where it has been shown that PR function can be associated to light-enhanced growth and survival, reduced respiration rate, and enhanced substrate uptake [4–7, 18–23]. However, these diverse functions are associated with the particular bacterial species studied, and sometimes even differ between strains, showing that rhodopsin phototrophy varies among bacteria in ways that are still difficult to predict from genomic and metagenomic data alone [24]. Therefore, studies combining physiology and molecular approaches on additional cultured bacteria are needed to further elucidate how PRs influence microbial physiology and ecological processes, such as nutrient processing rates and how these influence biogeochemical cycles and the marine microbial loop.

Recently, the presence of *pr* genes was reported in the *Verrucomicrobiota* phylum, based on metagenome-assembled genomes (MAGs) from seawater collected in the Western Mediterranean Sea [25]. *Verrucomicrobiota* are nearly ubiquitous in the marine environment, constituting on average 2% of microbial communities [26]. This phylum appears to have an important role in the cycling of high molecular weight compounds, showing an abundance of genes involved in degradation of carbohydrates, and especially sulfated polysaccharides [27**–**29]. *Verrucomicrobiota* are diverse and include both motile and non-motile members [30] that can live both as free-living and particle-associated microorganisms [31]. However, the absence of cultured photoheterotrophic *Verrucomicrobiota* strains has precluded the determination of PR functions in this group.

Here, we characterize the first two rhodopsin-bearing *Verrucomicrobiota* strains. The first strain, ISCC53^T^ (assigned here to *Candidatus* Pelagisphaera phototrophica gen. nov., sp. nov.), encodes a PR/retinal biosynthesis gene cluster and is related to the heterotrophic shallow-water genus *Pelagicoccus*. The second strain, ISCC51, harbors a xanthorodopsin-like gene and represents an undescribed *Opitutales* species, the first isolate of the family-level clade UBA2995. Using the genomic sequences of these isolates, we determined their global distribution and abundance. For ISCC53^T^ we tested which cellular processes are affected by rhodopsin function and determined how such processes are modulated by environmental conditions. Our findings suggest that rhodopsins support nutrient uptake, particularly under carbon-starved conditions, leading us to propose that photoheterotrophic *Verrucomicrobiota* occupy an ecological niche where light harvesting supports motility toward organic matter and nutrient transport into starved cells.

## Methods

### Adaptation of strains ISCC51 and ISCC53^T^ to a defined medium and genomes closure

The *Verrucomicrobiota* strains ISCC51 and ISCC53^T^ characterized here, were isolated in our previous study (isolates A3 and F3, respectively) by dilution to extinction cultivation technique in low-nutrient heterotrophic media (‘LNHM’) [32**–**34]. The previously obtained draft genomes were closed with additional PCR reactions and Sanger sequencing. To conduct controlled physiology experiments, we adapted the strains to a defined artificial seawater medium, here called ASM-1. The medium composition is provided in Supplementary File 1.

### Scanning electron microscopy

Cells used for morphological analysis were grown in ASM-1 amended with glucose (50 μM) and phosphate (KH_2_PO_4_, 50 μM) to mid-logarithmic phase (ISCC53^T^) and to stationary phase (ISCC51) before filtration on a 13 mm 0.2 μm polycarbonate membrane filter (Nuclepore, Whatman). Filters were processed as previously described [6] (details in Supplementary File 1). Samples were examined at 3 kV in a field emission Scanning Electron Microscope (CARL ZEISS SIGMA HD, Germany).

### Genome classification

Verrucomicrobial genome assemblies were collected from NCBI Assembly by retrieving all records assigned to *Verrucomicrobiota*/*Verrucomicrobia* in NCBI and the Genome Taxonomy Database (GTDB) r.202. Collected genome assemblies were classified with GTDBtk v.1.5.0 when absent from GTDB. Rough phylogenetic relationships between diverse groups of the phylum were visualized based on the GTDB backbone phylogeny with the phylogenetic placement of the additional assemblies by clustering assemblies on the level of pairwise tree distances of 0.1. Phylogeny of the order *Opitutales* (*sensu* GTDB) was reconstructed by creating species-level non-redundant set of assemblies with dRep v. 3.0.0 (-pa 0.9 -sa 0.95 -nc 0.30 -cm larger) [35] and analyzing it with phylophlan v. 3.0.2 (--diversity medium, concatenated multi-gene alignment, IQ-TREE 2 for phylogeny inference) [36]. Throughout the paper we utilize the GTDB classification for the phylum.

### Delineation of *Candidatus* Pelagisphaera

Affinities of the strain ISCC53^T^ and related assemblies were first assessed with GTDB and GTDBtk. Phylogenetic relationships between genomes assigned to GTDB sister genera g__UBA5691 and g__Pelagicoccus were reconstructed with phylophlan (--diversity low, concatenated multi-gene alignment, IQ-TREE 2 for phylogeny inference). Pairwise genome-wide average nucleotide identity (gANI) and alignment fraction values were calculated with dRep. Pairwise identities in the 16S rRNA gene were calculated as the number of matches divided by the sum of matches and mismatches in a Needleman-Wunsch alignment (gap open 16, gap extension 4, match 5, mismatch -4).

### Growth conditions for light versus dark comparisons

Strain ISCC53^T^ was grown in ASM-1 amended with glucose (15 μM) and KH_2_PO_4_ (2 μM). At this glucose concentration cells enter stationary phase as a result of carbon depletion (Supplementary Fig. S1). Cells were grown at 20 °C in darkness or 12:12 light:dark cycles (to represent natural diel cycles) using a blue-light emitting diode (LED, Aqua Illumination) set to 30 μmol photons m^−2^ s^−1^ as a light source. Cells growth was monitored using a flow cytometer (Guava EasyCyte Plus, Millipore) and samples were collected in logarithmic growth phase for the carbon-replete condition and in stationary phase for the carbon-deplete condition to run different measurements: retinal quantification, ATP production as response to light/dark shifts, and substrate-uptake rates (^3^H-glucose) (see method details below). For comparison, growth curves were also conducted in ASM-1 medium amended with KH_2_PO_4_ (2 μM) and a higher glucose concentration (50 μM), in darkness or 12:12 light:dark cycles (blue-emitting LED, 30 μmol photons m^−2^ s^−1^).

### Retinal quantification

Retinal concentrations were determined and used as a proxy for PR quantification using the method described elsewhere [9] with previously published modifications [37]. Details are provided in Supplementary File 1.

### ATP assays

Cells were exposed to eight successive periods of light and dark (5 min each) at which time points, samples were taken for ATP measurements. Cellular ATP concentrations were measured using a luciferase-based assay (BactTiter Glo, Promega, Madison, WI) as previously reported [6]. Details are provided in Supplementary File 1.

### ^3^H-Glucose uptake efficiency

Glucose was selected as substrate for testing substrate uptake efficiency, because it was shown to be used as carbon source for biomass accumulation (Supplementary Fig. S1). Cells were collected at logarithmic growth phase for the carbon-replete condition and at stationary phase for the carbon-deplete condition from both the dark and the light:dark treatments. Prior to radioactive assays, cells were concentrated by centrifugation (20,000 rpm, 16 °C, 1 h), washed in artificial seawater (ASW) and resuspended in ASW to reach cell densities of *ca*. 10^7^ cells/ml. Samples were exposed to either light (LED blue light at 130 µmol photons m^−2^ s^−1^) or darkness (covered in aluminum foil). “Kill samples” (dead cells, preserved with formalin 2% 1 h prior to the experiment) served as negative control. Glucose-[^3^H] (0.05 µM, final concentration, Perkin-Elmer) radioisotope uptake assays were conducted at 20 °C and samples taken after 30, 90, and 150 min of incubation. Radiolabeled glucose uptake was determined as follows: 700 µl of cells were collected in triplicates at each time point via filtration through a 25 mm GSWP 0.22 µm filter (Millipore) and filters were then rinsed 6 times with 2 ml aliquots of ASW to remove radioisotope excess. After rinsing, all filters were transferred to scintillation vials containing 5 ml UltimaGold (Perkin-Elmer) scintillation fluid and allowed to sit overnight prior to being read on a Tri-Carb 2810TR liquid scintillation counter (Perkin-Elmer). Difference between treatments were analyzed by analysis of variance (ANOVA) with Exposure (light/dark) and Carbon (replete/deplete) as between variables, and glucose uptake rate as the dependent variable.

### Spatiotemporal distribution of ISCC51 and ISCC53^T^ in the Eastern Mediterranean Sea based on 16S rRNA gene amplicon sequencing data

The relative abundance of ISCC51 and ISCC53^T^ at station N1200 along a depth profile was determined during six research cruises (twice each in spring, summer, and winter) for the free-living (0.22–5 µm) and the particle-associated (>11 µm) fractions. A detailed description of the samples and derived 16S rRNA gene datasets is provided in [38]. To examine the relative abundance of close relatives of ISCC51 and ISCC53^T^, their 16S rRNA gene sequences were aligned (Muscle algorithm in MEGA7) and cut at the same sites of the primers used in [38], resulting in sequences corresponding to positions 515–938 in *E. coli*. Assembled, quality-controlled reads from [38], were matched against the partial 16S rRNA gene sequences of the two isolates with BLAST using a threshold of 98% identity. The relative abundance in each sample was calculated as the number of matched reads divided by the total number of reads used in the BLAST search. An additional analysis testing for relative abundances of exact sequence variants (ESVs) with ≥98% sequence identity to the 16S rRNA gene of ISCC51 and ISCC53^T^ is described in the Supplementary File 1.

### Global distribution of *Ca*. Pelagisphaera and of its PR/retinal biosynthesis gene cluster

Global distribution of *Candidatus* Pelagisphaera and its PR/retinal biosynthesis gene cluster was assessed by analyzing the abundance of marker genes as well as genes for PR (*pr*) and the beta-carotene 15,15′-dioxygenase (*blh*), which encodes for the enzyme responsible for the last step of retinal synthesis [3], in the Tara Ocean Microbiome Reference Gene Catalag v.2 (OM RGC v.2) [39]. The marker genes (MGs) were selected from the set defined in [39]. OM-RGC v.2 records assigned to each of the 40 MG orthogroups were matched against verrucomicrobial protein sequences with usearch_global and those records that had best hits against *Ca*. Pelagisphaera with a protein identity value of ≥95% were selected. A set of 20 most reliable MGs was selected based on mutual correlation and outlier detection across Tara Oceans samples: COG0012, COG0016, COG0052, COG0080, COG0081, COG0087, COG0091, COG0093, COG0096, COG0098, COG0100, COG0124, COG0185, COG0197, COG0200, COG0215, COG0256, COG0495, COG0541, and COG0552. *Ca*. Pelagisphaera abundance was calculated as the mean abundance (i.e., per-base read coverage estimated with MOCAT, see [40]) of the chosen MGs per station or sample and the fractions of *Ca*. Pelagisphaera in the whole microbial community were obtained by dividing the *Ca*. Pelagisphaera abundances by the average total marker abundance. Catalog records with ≥95% identity to the *Ca*. Pelagisphaera *pr* and *blh* genes were searched for using the same strategy. Average copy numbers for *pr* and *blh* were obtained by dividing their abundances by the *Ca*. Pelagisphaera abundances obtained from MGs. The copy numbers were estimated for stations/samples in which ≥90% of the *Ca*. Pelagisphaera MGs had coverage above zero.

### Prediction, classification, and phylogenetic analysis of rhodopsin genes

ORFs in unannotated genomes were predicted with GeneMarkS v. 4.32 [41]. Rhodopsin genes were searched for with hmmscan from the HMMER package v.3.3.2 [42] against a custom HMM profile created from a curated database of diverse microbial rhodopsin subfamilies. Sequences passing an *E* value threshold of <1e−5 were then classified into rhodopsin subfamilies by searching them against a curated database of reference sequences with NCBI blastp v. 2.11.0 + [43]. Rhodopsin sequences from the subfamilies related to PRs and XRs were selected for downstream analysis. Additional reference rhodopsin sequences were retrieved from Uniprot and Uniparc r. 2021_02 and the combined sequences were clustered at 100% identity level with cdhit v. 4.8.1 [44]. To exclude short and divergent sequences, only sequences with the retinal-binding lysine residue in the transmembrane helix 7 were selected at this step. Outgroup sequences were added from unrelated rhodopsin families: ARII, ASR, BR, CR, GtR1, GtR2, HsHR, HsSRI, LR, NOP-1, NpSRII, OmR2, PaR, SyHR. The filtered unique rhodopsin sequences were sequentially clustered at 90%, 80%, 70%, and 60% identity levels, aligned with mafft v.7.505 (--localpair --maxiterate 1000) [45] and trimmed with trimAl v.1.4.1 (-automated1) [46]. Phylogeny was reconstructed with 10 independent runs of IQ-TREE 2 v.2.2.03 (-pers 0.2 -nstop 500) [47] selecting the run with the highest likelihood. To facilitate assignment of short sequences from verrucomicrobial assemblies, the original rhodopsin sequences were matched against the final set of the 60%-identity clusters with usearch_global as implemented in usearch v. 11.0.667 [48].

## Results

In a previous study, using Eastern Mediterranean Sea offshore seawater from the DCM depth as inoculum, we isolated *Verrucomicrobiota* strains harboring rhodopsin genes. Two strains, previously called A3 and F3 [33], received the strain codes ISCC51 and ISCC53^T^, respectively, and their draft genomes were closed in the current study to yield complete circular genomes of 4,815,746 bp and 4,086,264 bp, respectively. The genome of ISCC51 encodes a xanthorodopsin (XR) gene, whereas the genome of ISCC53^T^ encodes a PR gene.

### Morphological traits

**Fig. 1:**
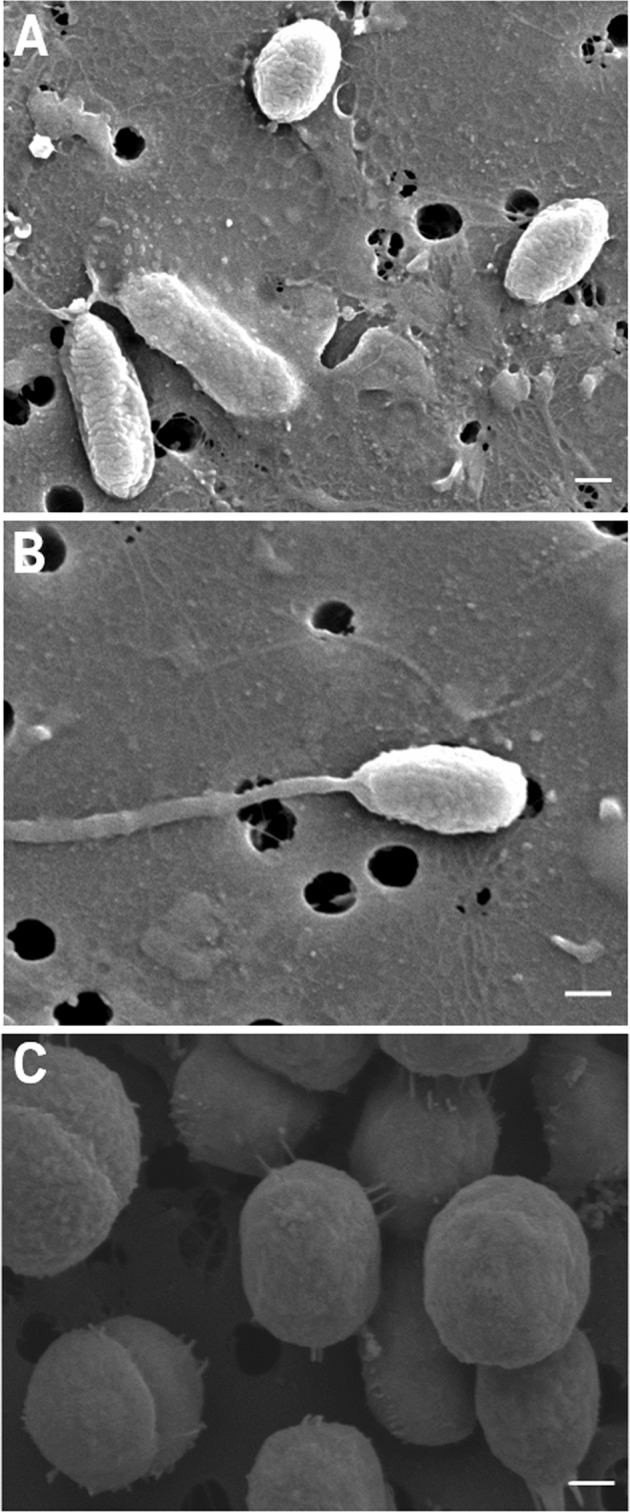
Morphology of photoheterotrophic *Verrucomicrobiota*. Scanning electron micrographs of (**A** and **B**) strain ISCC51 and (**C**) strain ISCC53^T^. Scale bar – 200 nm.

Morphological characteristics of ISCC51 and ISCC53^T^ were investigated using electron microscopy. ISCC51, the XR-containing isolate, had coccobacillus-like morphology (690 ± 88 nm length, 408 ± 32 nm width, *n* = 8) and some cells had polar flagella (Fig. 1A and B). By contrast, the cells of strain ISCC53^T^ appeared coccoid in shape (741 ± 76 nm in diameter, *n* = 12) with pili extending from the cells or connecting between cells; division appeared to be by binary fission (Fig. 1C). While flagellar genes were present in both genomes, no flagella were observed in the electron microscopy images of ISCC53^T.^

### ISCC53^T^ is the type strain of a new genus, *Candidatus* Pelagisphaera

**Fig. 2:**
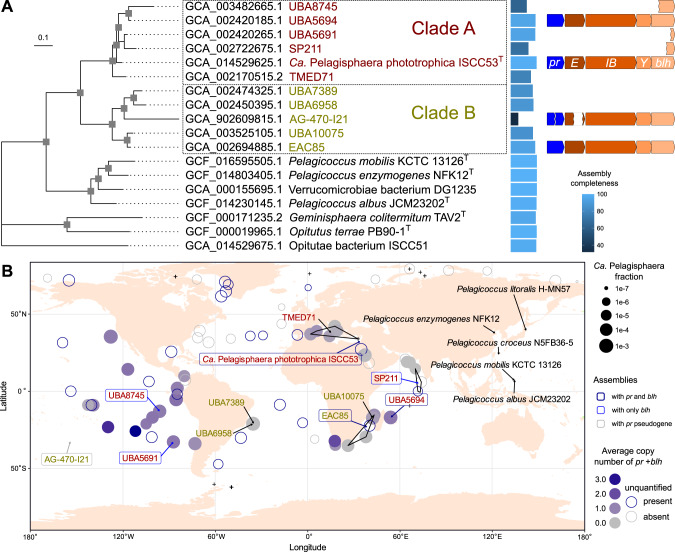
Phylogeny and distribution of *Candidatus* Pelagisphaera. **A** Multi-gene maximum likelihood species phylogeny of the *Ca*. Pelagisphaera/*Pelagicoccus* clade with genome completeness and PR gene cluster indicated. Among the *Ca*. Pelagisphaera only ISCC53^T^ is cultured, while all *Pelagicoccus* genomes are from cultured strains. Genomes from the two *Ca*. Pelagisphaera lineages (Clade A and B) are highlighted in red and green. The tree is outgroup-rooted. Nodes with fast bootstrap support values above 0.99 are highlighted. **B** Geographical distribution of *Ca*. Pelagisphaera marker genes and PR gene cluster based on the *Tara* Oceans data (OM-RGC v.2). Each dot represents a *Tara* Oceans station (SRF and DCM merged together) with the size proportional to the fraction of *Ca*. Pelagisphaera marker genes out of total microbial community and the color reflecting the estimated average copy number of the PR gene cluster (averaged over *pr* and *blh*). For stations at which the coverage for the marker genes was low only mere presence and absence of the PR gene cluster coverage is indicated. Provenance of the cultured isolates and MAGs is indicated.

Comparison of the genome assembly of ISCC53^T^ to related metagenome-assembled genomes (MAGs), single-cell amplified genomes (SAGs), and genomes of cultured isolates revealed it to be a member of a well-attested clade related to the genus *Pelagicoccus* (Fig. 2A, Supplementary Fig. S2). According to the criteria of GTDB, it was assigned to a genus-level taxon g__UBA5691. Thus far, the clade has been composed entirely of genomes from environmental samples making ISCC53^T^ its first cultured representative. Differences in genome alignment fraction, genome-wide average nucleotide and 16S rRNA gene sequence similarity between g__UBA5691 and *Pelagicoccus* fall well outside of the range observed within bacterial genera [49], implying that they represent two sister clades that differ on a level warranting the establishment of a new genus (Supplementary Fig. S3). In addition, ecological differences, such as biogeographic distribution, further distinguish these two genera. All of the *Pelagicoccus* strains have thus far been isolated from shallow waters of the west Pacific Ocean (Fig. 2B) and the metagenomic data from the *Tara* Oceans project (which does not include the west Pacific Ocean) yielded no MAGs or shorter fragments containing marker genes attributable to *Pelagicoccus*. In contrast to the limited geographical distribution of *Pelagicoccus*, g__UBA5691 shows a global oceanic distribution, as described in detail below. Finally, whilst full or partial *pr-bhl* gene clusters were found in the genomes of ISCC53^T^ and several additional closely related genomes within g__UBA5691, such genes were completely absent from the four sequenced *Pelagicoccus* genomes. Besides ISCC53^T^ itself, three more assemblies were found to contain the complete gene cluster, but one of them, SAG AG-470-I21, had deletions in the *pr* and *crtE* genes indicative of pseudogenization. Three additional assemblies contained scaffolds terminating with the 3′ end of the *blh* (β-carotene 15,15′-dioxygenase) gene indicating that at least part of the corresponding bacterial populations contained the gene cluster (Fig. 2A).

Given the significantly distinct genomic characteristics between g__UBA5691 and *Pelagicoccus*, we propose the new genus *Candidatus* Pelagisphaera [Pe.la.gi.sphae’ra. L. neut. n. *pelagus*, open sea; L. fem. n. *sphaera*, sphere: N.L. fem. n. *Pelagisphaera*, a sphere-shaped organism from the open sea] with ISCC53^T^ as its type strain. Based on the presence of a proteorhodopsin gene in the genome of strain ISCC53^T^ and its function in light energy capture (described below), we propose the new species to be called *Candidatus* Pelagisphaera phototrophica [pho.to.tro’phi.ca. Gr. neut. n. *phos, photos*, light; N.L. masc. adj. *trophicus* (from Gr. masc. adj. *trophikos*, nursing, tending or feeding); N.L. fem. adj. *phototrophica*, feeding on light, phototrophic]. *Ca*. Pelagisphaera subdivides into two well-supported lineages: clade A and clade B (Fig. 2A, Supplementary Fig. S3, Supplementary Table S1). A total of five species are delineated by the criteria of GTDB with most of the genomes assigned to the same species as *Ca*. P. phototrophica in clade A (Supplementary Table S1).

### ISCC51 is an example of *Verrucomicrobiota* with xanthorhodopsin

**Fig. 3:**
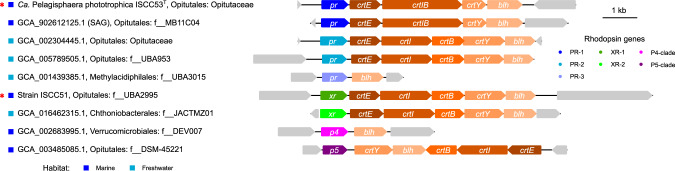
Rhodopsin gene clusters associated with different rhodopsin subfamilies. Clusters originate from MAGs or, in the case of ISCC51 and ISCC53^T^ (red asterisks), from cultured strains. *crtIB* is a putative fusion gene coding for bi-functional phytoene desaturase/phytoene synthase.

**Fig. 4:**
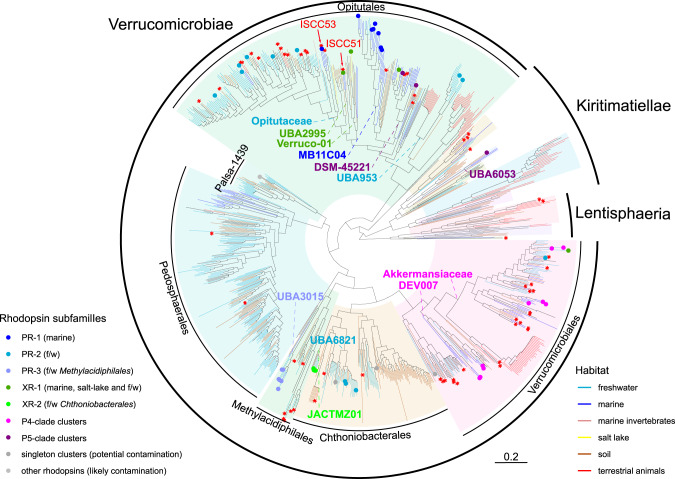
*Verrucomicrobiota* species phylogeny. Phylogeny is based on the backbone GTDB reference tree and phylogenetic placement of the additional assemblies. Redundant assemblies were removed (0.1 tree distance). Asterisks indicate assemblies coming from cultured strains. GTDB classes and orders are indicated. Generic and familial labels are provided when at least one assembly has a rhodopsin gene.

We previously demonstrated that the strain ISCC51 belongs to the family-level clade f__UBA2995 closely related to the family *Opitutaceae* that also includes two MAGs associated with the sponge *Petrosia ficiformis* [33] (see also Supplementary Fig. S2). ISCC51 is thus far the only cultured representative of this group and the only one encoding a rhodopsin gene. In contrast to the majority of the rhodopsin proton pumps from *Verrucomicrobiota*, the rhodopsin from ISCC51 belongs to the xanthorhodopsin subfamily (Fig. 3 and Supplementary Fig. S7). The *xr* genes similar to that of ISCC51 appear sporadically in unrelated *Opitutales* from diverse habitats and cluster among XRs from *Proteobacteria* (Fig. 4 and Supplementary Fig. S7).

### *Ca*. P. phototrophica ISCC53^T^ utilizes light for ATP production under carbon-starved conditions

**Fig. 5:**
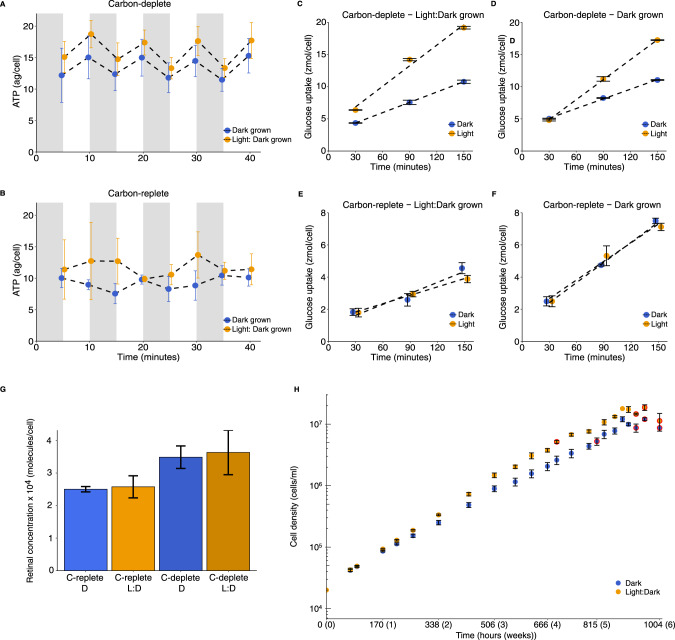
Physiological responses of *Ca*. Pelagisphaera phototrophica to light. **A**,**B** Cellular ATP content of carbon-starved (deplete) (**A**) and carbon-replete (**B**) cells 5 min after exposure to either dark (gray background) or light (white background) at eight time points. **C–F** Uptake of radio-labeled ^3^H-glucose (0.05 µM final concentration) by *Ca*. P. phototrophica cells in light (130 µmol photons m^−2^ s^−1^, yellow symbols) versus dark (blue symbols; mean ± s.d. of triplicate measurements) cultured in carbon-replete (**E**, **F**) and carbon-deplete (**C**, **D**) conditions under light:dark (12:12 h) cycles (**C**, **E**) and continuous dark (**D**, **F**). Standard deviations in (**C**–**F**) are based on three technical replicates (i.e., triplicate samples from the same culture). **G** Cellular retinal content in cells from the four investigated culture conditions (mean ± s.d. of triplicate cultures). **H** Growth curves of *Ca*. P. phototrophica under light:dark (12:12 h) cycles versus continuous dark conditions. Standard deviations in (**H**) were calculated from flow cytometry measurements taken on triplicate cultures. Red symbols show time points at which samples were taken for the physiological assays shown in (**A**–**G**). Cells taken at 694 and 815 h represent log phase samples (carbon-replete condition).

To determine the physiological function of PR, we took advantage of our *Ca*. P. phototrophica isolate and compared its growth under light:dark cycles versus continuous darkness when grown with 15 µM glucose. The faster growth in light:dark cycles as compared to continuous darkness was only observed at this low carbon concentration (15 µM glucose, Fig. 5H). When grown at higher glucose concentrations (50 µM glucose), no difference in the growth curves under light:dark versus dark was observed (Supplementary Fig. S4).

Light-dependent energy production was tested in cultures grown with 15 µM glucose, under constant darkness or under light:dark (12:12 h) cycles at both logarithmic and stationary phase by measuring cellular ATP concentrations after 5 min intervals of dark and light (130 µmol photons m^−2^ s^−1^) exposure. The interaction between carbon (replete/deplete) and light-dark exposure (after dark/after light) was significant (*F*(1,2) = 42.37, MSE = 1.24, *p* = 0.002). Our results show that in stationary phase (carbon-deplete condition) cellular ATP levels increased significantly in response to light exposure (*F*(1,2) = 41.36, MSE = 3.28, *p* = 0.003) regardless of the culture having been previously grown in dark or in light:dark conditions (Fig. 5A). Conversely, cultures in logarithmic phase (carbon replete condition), both when previously grown in light:dark and in continuous dark conditions, did not present significant differences in their ATP levels when exposed to intervals of light or dark (*F*(1,2) = 1.26, *p* = 0.32) (Fig. 5B).

Since proteorhodopsin-mediated *pmf* has previously been shown to power ATP-mediated substrate uptake [6], we tested whether the ATP derived from the proton-pumping activity of the proteorhodopsin is used for phosphorylation-dependent substrate uptake. For this, we analyzed ^3^H-glucose uptake of both light- and dark-grown cultures, in carbon-replete (logarithmic growth) and in carbon deplete (stationary phase) cultures under either dark or light conditions (Fig. 5 C–F). Carbon-deplete cells showed higher glucose uptake rates during light exposure versus darkness, regardless of having been previously grown under light:dark (12:12 h) cycles (*F*(1,8) = 960.13, MSE = 0.000006, *p* < 0.5 × 10^–6^) or in darkness (*F*(1,8) = 890.24, MSE = 0.000005, *p* < 0.5 × 10^–6^) (Fig. 5C,D). By contrast, for carbon-replete cells, no differences in glucose uptake rates were observed between light and dark for dark grown cells (*F* = 2.59, *p* = 0.14), while a minor, yet significant (*F*(1,8) = 7.25, MSE = 0.000006, *p* < 0.05) higher uptake rate was found in the dark versus light condition, for the cells grown under light:dark cycles (Fig. 5E,F).

The previous experiments suggest that PR functions are regulated by carbon availability. We thus analyzed cellular retinal concentrations in carbon-replete and carbon-deplete *Ca*. P. phototrophica grown both under light:dark and continuous darkness conditions. Cellular retinal concentrations were not affected by light exposure, with 31,046 ± 10,174 retinal molecules/cell estimated for the light-adapted cells compared to 29,955 ± 6651 retinal molecules/cell for the dark-adapted cells (F < 1) (Fig. 5G). Cellular retinal concentrations were higher in carbon-deplete (35,601 ± 8441 retinal molecules/cell) compared to carbon-replete conditions (25,401 ± 3846 retinal molecules/cell), however, this difference failed to reach statistical significance (*F*(1,4) = 4.25, MSE = 294.91, *p* = 0.1). A larger scale experiment is required to test whether this trend reflects regulation of retinal synthesis by substrate availability in *Ca*. P. phototrophica.

Parallel experiments run on ISCC51 could not determine the conditions under which this strain would enter stationary phase in response to carbon depletion. Different glucose concentrations in the medium resulted in different growth rates, but the maximum cell yields were the same. Thus, lower glucose concentrations provided a carbon limited rather than a carbon-deplete condition, and below a certain glucose threshold, ISCC51 would not grow. No light-mediated enhancement in cellular ATP levels was observed for neither carbon-replete nor carbon-limited ISCC51 cells (data not shown), therefore the conditions under which these bacteria benefit from the xanthorhodopsin remain to be elucidated.

### Depth and seasonal distribution of *Ca*. P. phototrophica and ISCC51 in the Eastern Mediterranean Sea

**Fig. 6:**
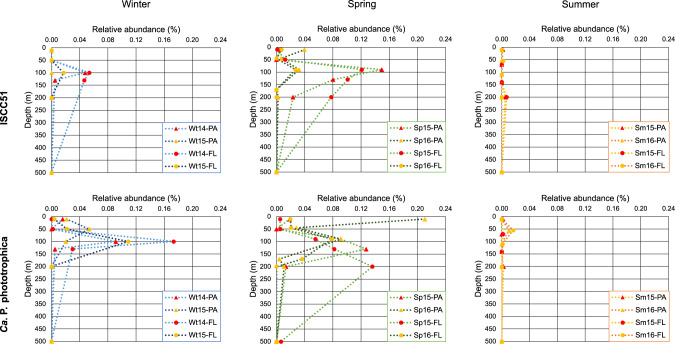
Spatiotemporal distribution of ISCC51 and *Ca*. P. phototrophica in the Eastern Mediterranean Sea. Relative abundance of ISCC51 and *Ca*. P. phototrophica in the particle-attached (PA, > 11 µm, triangles) and free-living (FL, 5–0.22 µm, circles) fractions at station N1200 based on 16S rRNA gene mapping (see methods for details). Samples were taken along a depth profile in two consecutive winters (Wt, blue lines), springs (Sp, green lines), and summers (Sm, orange lines) between 2014 and 2016 (last two digits of the year are given). The first three depths represent 10 m, half the depth of the DCM, and the DCM (between 90 and 130 m).

To gain additional insights into the potential ecological roles of *Ca*. P. phototrophica and ISCC51, we used a previously published 16S rRNA gene amplicon dataset [38] from the same Eastern Mediterranean Sea station, from which the strains were isolated. We calculated relative abundances of bacteria with >98% 16S rRNA gene sequence identity to *Ca*. P. phototrophica and to ISCC51 to elucidate how they vary with depth and by season. Close relatives of ISCC51 and *Ca*. P. phototrophica were mainly present in the water column in winter and spring, while they were almost absent in summer (Fig. 6, Supplementary Fig. S5). This suggests that summer water column stratification, which exacerbates the already ultraoligotrophic conditions of the Eastern Mediterranean Sea, results in a major reduction of the photoheterotrophic *Verrucomicrobiota* population. Their maximal relative abundances were mostly found at 100 m depth, which corresponds to the depth at which they were isolated. Both isolates were found not only as free-living, in the 0.22–5 µm fraction, but also as particle attached, in the 5–11 µm fraction (Fig. 6), suggesting an involvement in carbon sequestration via sinking particles. An additional analysis of individual exact sequence variants (ESVs) revealed that the two isolates (ISCC51 and *Ca*. P. phototrophica) represent the dominant variants at the N1200 station (Supplementary Fig. S5). Details on the ESV analysis are provided in Supplementary File 1.

### Global distribution of *Ca*. Pelagisphaera and of its PR/retinal biosynthesis gene cluster

After having characterized the physiological role of verrucomicrobial proteorhodopsins and analyzed the distribution patterns of *Ca*. P. phototrophica in the Eastern Mediterranean, we looked at the global distribution of *Ca*. Pelagisphaera and of its *pr* and *blh* genes. *Ca*. Pelagisphaera marker genes recruited significant coverage (at least 90% of the marker genes) at *Tara* Oceans stations from tropical and temperate waters with hotspots in the Pacific, Indian Ocean, and the Mediterranean (Fig. 2B). The analysis of *Ca*. Pelagisphaera marker, *pr* and *blh* genes showed the existence of locations with high abundance of *Ca*. Pelagisphaera, where coverage of the *pr-blh* gene cluster approaches zero, in the Atlantic and Indian Oceans. In other locations (East Pacific, Mediterranean, and off Africa’s southern coast) the average copy number of *pr-blh* per genome was close to one or even higher, reaching on rare occasions, >1.5 operon copies per genome (Fig. 2B), which can be explained either by duplications of the operon or horizontal gene transfer to other microbes. A depth distribution analysis comparing samples from surface, DCM, and mesopelagic layers from the *Tara* Oceans dataset, showed that *Ca*. Pelagisphaera was detectable at multiple depths, and demonstrated a trend of having higher incidence of *pr* and *blh* genes in the surface layer (Supplementary Fig. S6). These data are in agreement with the light-related function of proteorhodopsins and imply that *Ca*. Pelagisphaera can be either photoheterotrophic or strictly heterotrophic, depending on environmental conditions.

### Types and distribution of rhodopsins across the *Verrucomicrobiota* phylum

To provide a global overview on the types and distribution of rhodopsins across the whole *Verrucomicrobiota* phylum, we did a search for rhodopsin genes across 2916 genome assemblies. This yielded hits against multiple rhodopsin families in 215 genomes (Fig. 4). The rhodopsins were classified by family and clustered together at 60% identity level. This way, the diversity was reduced by tagging rhodopsin families appearing in less than three assemblies as likely contamination and clusters appearing only once as potential contamination. This conservative procedure retained verrucomicrobial rhodopsin sequences from four rhodopsin clades, all belonging to the superclade that unites XRs, PRs, and NQ pumps: PRs in 149 assemblies, an unnamed and uncharacterized clade that we refer to as P4 in 43 assemblies, XR-like in 14 assemblies and a different uncharacterized family that we refer to as P5, in five assemblies (Fig. 4, Supplementary Fig. S7). A detailed description of these diverse verrucomicrobial rhodopsins is provided in Supplementary File 1. Despite the widespread appearance of rhodopsin genes among *Verrucomicrobiota*, none of them could be found in any genome of cultured *Verrucomicrobiota* (Fig. 4), making *Ca*. P. phototrophica ISCC53^T^ (with a *pr* gene) and ISCC51 (with a *xr* gene) the first such cases.

Most rhodopsin-possessing *Verrucomicrobiota* harbor the retinal biosynthesis gene cluster next to the corresponding rhodopsin genes (*crtE*, *crtI*, *crtB* (or *crtIB*), *crtY*, and *blh*) and share a similar gene order with other prokaryotic groups (Fig. 3) [3, 50, 51]. The distinctive feature of the verrucomicrobial *pr* operons of the type seen in *Ca*. P. phototrophica ISCC53^T^ (PR-1) is the fusion of *crtI* (phytoene desaturase) and *crtB* (phytoene synthase). While some fusions of enzymes involved in carotenoid biosynthesis have been previously characterized, including natural CrtBY and CrtIBY, and synthetic fusions of the three enzymes [52, 53], this is the first report of a natural CrtIB fusion enzyme.

## Discussion

Despite the earlier reports about the presence of rhodopsin genes in assembled genomes of freshwater [54, 55] and marine [25, 56] *Verrucomicrobiota*, photoheterotrophy in this phylum has thus far remained largely unexplored. Our study thus provides the first systematic analysis of this phenomenon based on the two *Opitutales* isolates from the Eastern Mediterranean Sea encoding for a *pr* and an *xr* gene, respectively.

The photoheterotrophic *Verrucomicrobiota* strains analyzed in this study (ISCC51 and *Ca*. P. phototrophica (ISCC53^T^)) had been originally isolated in winter [33]. Furthermore, our analysis of the 16S rRNA gene datasets of six cruises from the Eastern Mediterranean Sea [38, 57] confirmed the presence of these verrucomicrobial photoheterotrophs in the more productive settings of winter and spring, with almost complete disappearance during the summer months (Fig. 6). Interestingly, in a different metagenomic study from the Mediterranean, a rhodopsin gene assigned to a verrucomicrobial MAG (MED-G86) recruited read coverage in a mixed winter water column and not from a stratified water column [25]. Taken together, the previous and present study suggest that photoheterotrophic *Verrucomicrobiota* may be unable to thrive in the extreme oligotrophic conditions typical of the stratified summer Mediterranean seawater. Instead, they may recurrently appear after winter deep-water mixing, being most abundant in the DCM layer.

Beyond the rhodopsin genes found in the isolates, the examination of 2916 verrucomicrobial genome assemblies revealed that approximately 7% might be photoheterotrophic, which highlights the fact that our strains do not represent isolated cases. Phylogenetic analysis shows divergent origins for verrucomicrobial rhodopsins, with the vast majority (72%) being PRs, while others come from the rarer subfamilies of P5, XR, and P4. Sequence analysis of two previously unreported rhodopsin clades found in *Verrucomicrobiota*, P4 and P5, indicates that they likely also function as proton pumps. A distinct feature observed here for the first time, was the fusion of the retinal biosynthesis genes *crtI* (phytoene desaturase) and *crtB* (phytoene synthase). Fusion of enzymes involved in the carotene biosynthetic pathway was shown to optimize metabolite transfer between the enzymatic reactions and reduce accumulation of intermediates, leading to increase in pathway efficiency [53].

Whilst *Ca*. Pelagisphaera has a global distribution, the photoheterotrophic genes show a patchy distribution across geographic locations in this group. Pseudogenization, as witnessed in one of the genomes affiliated with *Ca*. Pelagisphaera (Fig. 2), and suspected gene duplications may drive such ecological and genomic diversification among the different lineages. Loss of the photoheterotrophic genes may relate to a switch from an energy-limiting to an energy-rich niche. The existence of niche differentiation among sympatric *Ca*. Pelagisphaera is hinted at by the contrasting distribution of the two dominant *Ca*. Pelagisphaera 16S rRNA gene variants among the two fractions in the amplicon data (Supplementary Fig. S5B).

To connect the distribution of verrucomicrobial photoheterotrophs with function, we provided here the first ecophysiological characterization of the *Ca*. P. phototrophica PR, which is representative of PRs encountered in marine *Opitutales* in general (type PR-1, Fig. 4, Supplementary Figs. S2 and S7). We have shown that the light-mediated proton gradient translates into increased production of ATP in *Ca*. P. phototrophica only when the cells are carbon-starved, coinciding with the highest number of rhodopsin molecules per cell. Analogously, under carbon starvation, light stimulates higher ^3^H-glucose uptake rates. Light-stimulated growth is only observed at growth-rate limiting carbon concentration of 15 µM glucose, it is not detected at the higher carbon concentration of 50 µM glucose. We therefore conclude that only when exogenous carbon is the limiting factor, PR photoheterotrophy provides a tangible energetic benefit.

It can be speculated that in their natural environment, light exposure may enable these photoheterotrophs to maintain optimal substrate uptake rates under carbon-deplete conditions, enabling them to respond quickly to sporadic carbon inputs. Similarly to our observations, light was found to positively affect growth in a medium that was low in labile organic matter in specific flavobacterial strains from the genus *Dokdonia* [4], in which light may support enhanced *pmf*-dependent vitamin uptake [5]. An even closer analogy can be drawn with the oligotrophic *Ca.* Pelagibacterales (SAR11 clade), which also showed higher substrate uptake rates in the light only under carbon-deplete conditions [6]. Nevertheless, SAR11 did not show a light-stimulated growth response in any carbon condition tested [6, 58]. Compared to *Ca*. P. phototrophica, SAR11 bacteria have much smaller genomes (about one fourth in size) and lack motility capacity. Thus, while in SAR11 PR phototrophy may serve mostly to maintain the minimal energy levels to sustain survival under carbon starvation and to support substrate uptake when carbon becomes available again, in *Verrucomicrobiota* it may support additional processes, such as energizing flagellar movement to enable carbon-starved cells to reach suspended particles. Support for this hypothesis is further lent by the finding of *Verrucomicrobiota* related to ISCC51 and to ISCC53^T^ not only in the free-living microbial fraction, but also in the particle-associated fraction (on 11 µm filters), suggesting a dual lifestyle that also involves interaction with particles (Fig. 6). The potential for PR-mediated *pmf* to fuel flagellar motion, has indeed been shown experimentally in *Escherichia coli* expressing heterologous PR [59]. Here we observed flagella by SEM imaging in ISCC51, a strain that also showed a dual lifestyle (free-living and particle-associated), potentially enabling it to move between particles.

A recent study along the subtropical frontal zone off New Zealand showed that, although microbial rhodopsins are generally more abundant in the picoplankton size fraction (0.2–3 μm) that represents free-living microbes, at times, the larger (>3 μm) size fractions, containing particle-attached prokaryotes, dominate the rhodopsin signal [37]. Marine particles can contain recalcitrant compounds, such as fucose-containing sulfated polysaccharides, that only few taxa can degrade, thus promoting carbon sequestration into deeper water [60]. *Verrucomicrobiota* represent one such taxon and are suggested to be important contributors to polysaccharide degradation, in particular hydrolysis of sulfated polysaccharides [29, 56, 61, 62]. The photoheterotrophic verrucomicrobial strains studied here are no exception in this respect: their genomes encode for an arsenal of enzymes involved in carbohydrate degradation, with a prominent expansion of sulfatase genes (192 genes in *Ca*. P. phototrophica). Accordingly, rhodopsin activity may both support the high energetic costs involved in the biodegradation of complex polysaccharides and enable carbon-starved *Verrucomicrobiota* to reach these recalcitrant sinking particles. We thus hypothesize a new link between rhodopsin photosystems and degradation of marine particles made of refractory organic matter, which would result in a reduction in carbon sequestration *via* sinking particles. We suggest this hypothesis warrants further investigation, by future measurements of a potential light-enhanced flagellar motility and enzymatic activity in particle-associated rhodopsin-containing *Verrucomicrobiota* such as *Ca*. Pelagisphaera and ISCC51.

## Supplementary information

Supplementary File 1

## Data Availability

The closed genomes of *Ca*. P. phototrophica ISCC53^T^ and Opitutales bacterium ISCC51 are available from NCBI Genebank under accession CP076039.1 and CP076041.1, respectively (NCBI assemblies GCA_014529625.2 and GCA_014529665.2).

## Code availability

The code used for the bioinformatic analyses is available from https://github.com/BejaLab/pelagisphaera. Raw phylogenetic trees, results of species delineation analysis and other data are available from the Figshare repository 10.6084/m9.figshare.21126829. In the future, strains ISCC51 and ISCC53 will be available from the DSMZ public culture collection. Until then, we will provide them to anyone interested without any condition.
