# Supplementary material for: Rhodopsin-mediated nutrient uptake by cultivated photoheterotrophic Verrucomicrobiota
Source: ISME J. 2023 Apr 29;17(7):1063–73. doi: 10.1038/s41396-023-01412-1 (PMC10284914; doi:10.1038/s41396-023-01412-1)
Supplement: Supplementary file 1 [file 41396_2023_1412_MOESM1_ESM.pdf]

Rhodopsin-mediated nutrient uptake by cultivated photoheterotrophic *Verrucomicrobiota*

Rinat Bar-Shalom, Andrey Rozenberg, Matan Lahyani, Babak Hassanzadeh, Gobardhan Sahoo, Markus Haber, Ilia Burgsdorf, Xinyu Tang, Valeria Squattrito, Laura Gomez-Consarnau, Oded Béjà, Laura Steindler

Supplementary file S1

Supplementary Methods

Composition of ASM-1 medium

Artificial seawater (ASW, NaCl (481 mM), $MgCl_2\cdot 6H_2O$ (27 mM), $CaCl_2\cdot 2H_2O$ (10 mM), KCl (9 mM), $NaHCO_3$ (6 mM), $MgSO_4\cdot 7H_2O$ (28 mM)) was amended with the following compounds: NH_4SO_4 (400 μM), KNO_3 (2 μM), $FeCl_3$ (117 nM), NH_4Cl (5 μM), trace metals ($MnCl_2\cdot 4H_2O$ (9 nM), $ZnSO_4\cdot 7H_2O$ (800 pM), $CoCl_2\cdot 6H_2O$ (500 pM), $Na_2MoO_4\cdot 2H_2O$ (300 pM), Na_2SeO_3 (1 nM), $NiCl_2\cdot 6H_2O$ (1 nM)), methionine (5 μM), glycine (5 μM), pyruvate (1 μM) and 0.1% vitamin mixture (as per [1]); ASM-1 was then amended with different concentrations of glucose as carbon source and KH_2PO_4 (according to experiment).

Preparation of filter for SEM analysis

Filters were rinsed in a graded series of ASM-1 medium with distilled water (75%, 50%, 25%, 0% ASM-1) for 5 minutes each (to remove the salt), then dehydrated in a graded series of ethanol with distilled water (10%, 25%, 50%, 75% ethanol) for 10 minutes each. Just before critical point drying, samples were passed to ethanol 100% for 10 minutes. Critical point drying was performed

with liquid CO_2, after which samples were sputter coated with gold for 60 seconds (thickness of 5.8 nm) and mounted on stubs.

Retinal measurements

Cells were filtered on 0.22 µm Durapore filters and flash frozen in liquid nitrogen, then kept at -80 ºC until extraction. Filters were sonicated in 3 ml of liquid chromatography-mass spectrometry (LC-MS)-grade methanol on ice and in the dark for 30 s. Butylated hydroxytoluene (1%; 250 µl) was subsequently added, and the samples were left to extract for 24 hours at -20 ºC. Deuterated all-*trans*-retinal (final concentration 10 nM, 100 µl) was added as an LC-MS/MS internal standard to the sample extract followed by hydroxylamine (1 M; 100 µl). The sample extract was subsequently incubated under light for 2 hours. Retinal concentration was determined as total retinal oxime, which is the transformation product of reacting retinal (either *cis* or all-*trans* isomer) with hydroxylamine, using LC-MS (triple quadrupole LC-MS/MS).

ATP assay

Twenty µl of culture (of known cell density) were dispensed into 90 microliters of BactTiterGlo reagent in white 96-well assay plates (White w/Lid, Tissue Culture-Treated, BD Biosciences, San Jose, CA); one minute later luminescence was measured using a multi-function plate reader (High Sensitivity Microplate Luminometer (Berthold Technologies GmbH & Co. KG, Centro XS3 LB 960, Bad Wildbad, Germany) with a 1 s integration time. An ATP standard curve was used to transform RLU values into ATP concentration levels. The assay was performed as follows: 3 ml of cell suspension were placed in 5 ml glass vials (a water bath set at 20 ºC was used to minimize heat transfer from the light source to the samples). The light source was a blue LED at an intensity

of 130 μmol photons m^{-2} s^{-1}. Every 5 minutes the light was turned on or off at which point samples were taken for ATP measurements. Statistical analysis: we performed an analysis of variance (ANOVA) including the independent variables Carbon (replete/deplete), Light-Dark exposure (after dark/after light), and shift (first, second, third, fourth measurement) as within variables, Growth (light:dark/dark) as a between variable, and cellular ATP content as the dependent variable.

Analysis of exact sequence variants (ESVs)

To examine the relative abundance of close relatives of ISCC51 and ISCC53^T across a previously published 16S rRNA gene dataset [2], their 16S rRNA gene sequences were used to match the partial 16S rRNA gene sequences (V4-V5 region) from the dataset with blastn from the NCBI BLAST v. 2.13.0 [3]. The relative abundances of exact sequence variants (ESVs) with sequence identity ≥98% to ISCC51 and ISCC53^T in each sample were calculated as the total number of matched read pairs divided by 10,000 (the number of fragments chosen for subsampling). Haplotype networks of the ESVs with ≥95% identity to ISCC51 and ISCC53^T, as well as reference V4-V5 sequences were built with pegas v. 1.1 [4]. Notice that some of the originally reported ESVs were merged after adjusting the primer trimming.

Supplementary Results

Ca. Pelagisphaera lineages

Ca. Pelagisphaera subdivides into two well-supported lineages: the lineage of *Ca*. P. phototrophica includes two or three species-level groups according to the genome pairwise comparisons (gANI ≥ 96.5%, alignment fraction ≥ 60% [5] that are nevertheless mutually non-monophyletic, and the

second lineage includes three monophyletic species-level groups of genome assemblies (Figure 2, Figure S3). No geographical pattern in the distribution of the two lineages could be discerned (Figure 3).

Analysis of exact sequence variants (ESVs) matching ISCC51 and ISCC53^T

When analyzing the relative abundance of close relatives of ISCC51 and ISCC53^T at station N1200 along a depth profile and across six research cruises (dataset from [2]), we found that the two isolates represent the dominant variants at this station (Figure S5). Nevertheless, among the ESVs assigned to *Ca*. Pelagisphaera, yet another variant (A02) had systematic appearance across cruises alongside the ESV corresponding to ISCC53^T (A01) (Figure S5A and B). Despite a ≤1% difference in the V4-V5 region of their 16S rRNA sequences, the two ESVs showed significant differences in their tendency to appear either in the particle-associated fraction (A01) or free-living fraction (A02) (Fisher's exact test p < 1e-22). This might indicate a niche differentiation among closely related *Ca*. Pelagisphaera lineages, although further interpretation is difficult due to lack of complete genomes corresponding to the A02 variant. Minor ESVs closely related (≥98% V4-V5 sequence similarity) to ISCC51 showed only sporadic appearance across cruises, with the major ESV D01 (identical to ISCC51) appearing mainly but not exclusively in the free-living fraction (Figure S5C and D).

Types and distribution of rhodopsins across the *Verrucomicrobiota* phylum

While the PRs identified in *Verrucomicrobiota* did not form a monophylum, there was a clear pattern in the distribution of the three dominant PR clusters (Figure 5, Figure S2 and S7). The PR-1 cluster was found strictly among marine *Opitutales*, including *Ca.* Pelagisphaera. This cluster

includes proteobacterial sequences as well and in fact belongs to a PR clade dominated by *Proteobacteria*. In contrast, the cluster PR-2 appeared among various freshwater verrucomicrobial lineages in *Chthoniobacterales* and *Opitutales*, in particular the multiple genera of the family *Opitutaceae* (*sensu* GTDB). This cluster is similarly nested among Proteobacteria-dominated clusters (Figure S4). At last, cluster PR-3 is restricted to the freshwater *Methylacidiphilales* and appears to belong to a Bacteroidota-dominated PR clade (Figure S7).

Rhodopsin clade P4 was found in multiple genome assemblies in *Verrucomicrobiales*, while P5 had a very sporadic appearance among *Opitutales*, as well as the *Kiritimatiellae* (Figure 5). The two clades are related and together with several other rhodopsin clades appear at the base of the superclade that unites PRs, XRs and NQ pumps (Figure S7). The transmembrane helix 3 (TM3) motifs observed in P4 and P5 are restricted to DTE (P4) and DTK (P4 and P5), indicative of a proton-pumping activity as the DTE motif is typical in characterized members of both, the PR and the XR subfamilies [6], and DTK is the motif found in the proton pump ESR [7]. Clade P4 as a whole has a very distinct pattern of appearance, being restricted to unrelated lineages in the PVC phyla *Planctomycetota* and *Verrucomicrobiota* (Figure S7).

XRs (or XR-like rhodopsins) have a very modest appearance among *Opitutales* (cluster XR-1), including strain ISCC51, *Verrucomicrobiales* (XR-1) and *Chthoniobacterales* (XR-2) (Figure 5, Figure S2).

Supplementary Table

Table S1. Genomes assigned to the genus *Ca*. Pelagisphaera (= g__UBA5691 in GTDB). "Clade" attributes each genome to one of the two clades within the genus (see Figure 2A and Figure S3). The clades are further subdivided into five species according to GTDBtk analysis (the species are additionally numbered in parentheses for clarity). Abbreviations: CG – complete genome, MAG – metagenomically assembled genome, SAG – single cell amplified genome, IO – Indian Ocean, MED – Mediterranean Sea, SAO – South Atlantic Ocean, SPO – South Pacific Ocean.

Assembly	Name	Type	Origin	Clade	GTDBtk species	Com-pleteness	Conta-mination
GCA_014529625.1	*Ca.* P. phototrophica ISCC53^{T}	CG	MED	A	sp002420265 (1)	98.78	0.65
GCA_002170515.2	TMED71	MAG	MED	A	sp002420265 (1)	77.26	1.14
GCA_002420265.1	UBA5691	MAG	SPO	A	sp002420265 (1)	93.83	1.02
GCA_002722675.1	SP211	MAG	IO	A	sp002420265 (1)	67.76	0.41
GCA_002420185.1	UBA5694	MAG	IO	A	sp002420185 (2)	94.69	0.85
GCA_003482665.1	UBA8745	MAG	SPO	A	sp002420185 (2)	61.33	1.63
GCA_902609815.1	AG-470-I21	SAG	SPO	B	(3)	28.95	0
GCA_002694885.1	EAC85	MAG	IO	B	sp002694885 (4)	93.47	1.59
GCA_003525105.1	UBA10075	MAG	IO	B	sp002694885 (4)	84.94	1.67
GCA_002450395.1	UBA6958	MAG	SAO	B	sp002474325 (5)	86.93	1.26
GCA_002474325.1	UBA7389	MAG	SAO	B	sp002474325 (5)	84.26	1.06

Supplementary Figures

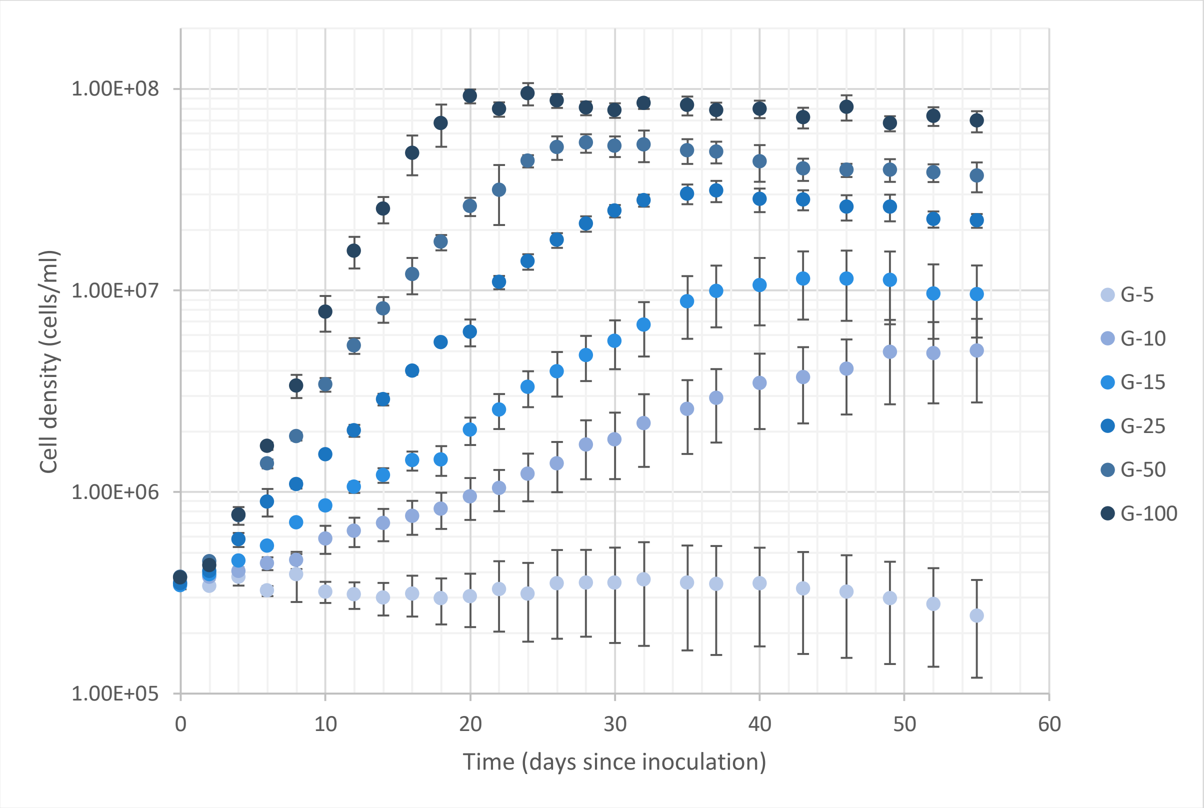

Figure S1. Growth curves of *Ca.* Pelagisphaera phototrophica grown in ASM-1 medium amended with KH_2PO_4 (2 µM) and with different glucose (G) concentrations (5-100 µM glucose). Average and standard errors are shown for triplicate cultures (with the exception of the condition glucose 50 µM, where only two replicates were available).

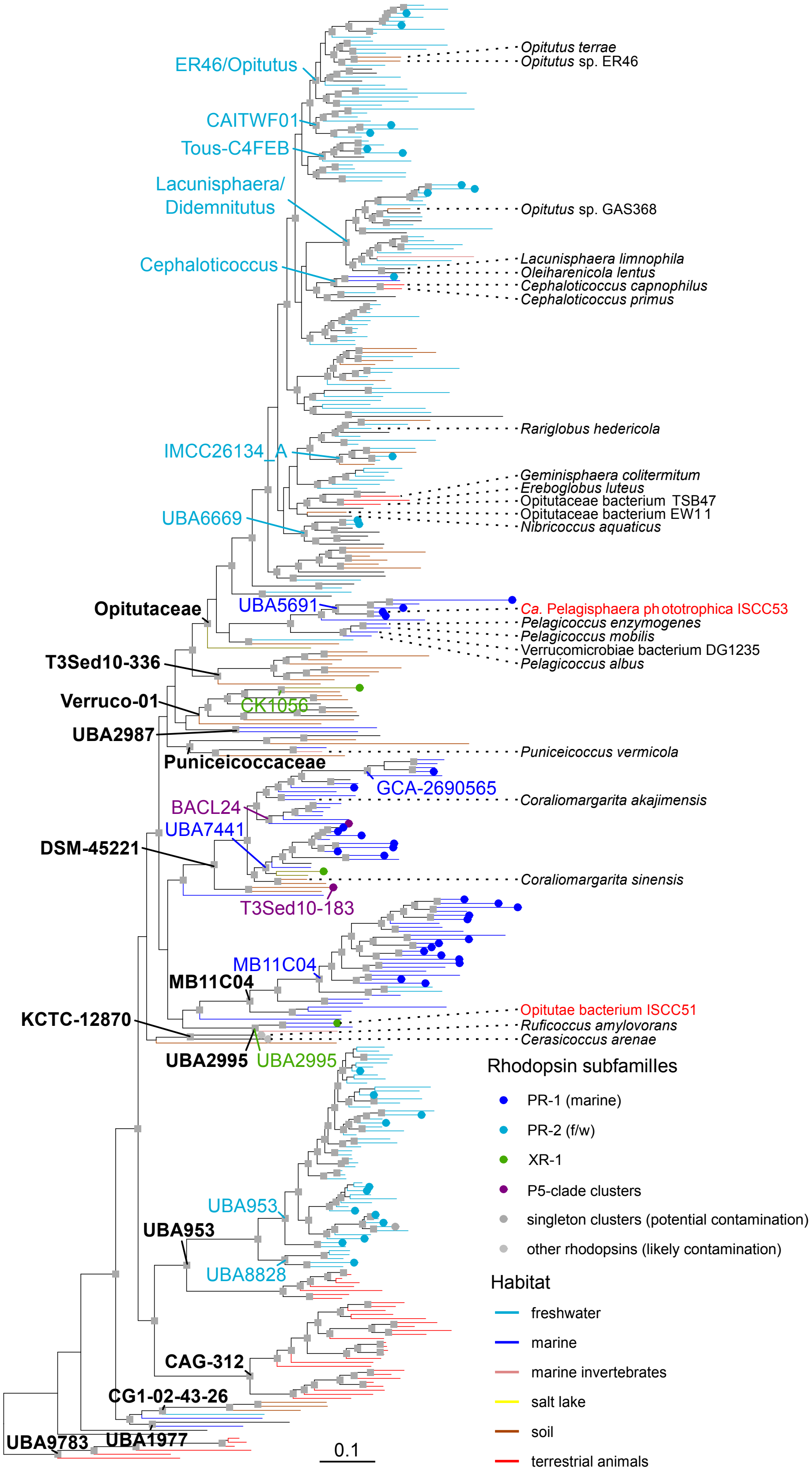
ER46/Opitutus
CAITWF01
Tous-C4FEB
Lacunisphaera/
Didemnitutus
Cephaloticoccus
IMCC26134_A
UBA6669
Opitutaceae
UBA5691
T3Sed10-336
Verruco-01
CK1056
UBA2987
Puniceicoccaceae
GCA-2690565
BACL24
UBA7441
DSM-45221
T3Sed10-183
MB11C04
MB11C04
KCTC-12870
UBA2995
UBA2995
UBA953
UBA953
UBA8828
CAG-312
CG1-02-43-26
UBA9783
UBA1977
0.1
Opitutus terrae
Opitutus sp. ER46
Opitutus sp. GAS368
Lacunisphaera limnophila
Oleiharenicola lentus
Cephaloticoccus capnophilus
Cephaloticoccus primus
Rariglobus hedericola
Geminisphaera colitermitum
Ereboglobus luteus
Opitutaceae bacterium TSB47
Opitutaceae bacterium EW11
Nibricoccus aquaticus
Ca. Pelagisphaera phototrophica ISCC53
Pelagicoccus enzymogenes
Pelagicoccus mobilis
Verrucomicrobiae bacterium DG1235
Pelagicoccus albus
Puniceicoccus vermicola
Coraliomargarita akajimensis
Coraliomargarita sinensis
Opitutae bacterium ISCC51
Ruficoccus amylovorans
Cerasicoccus arenae
Rhodopsin subfamilles
PR-1 (marine)
PR-2 (f/w)
XR-1
P5-clade clusters
singleton clusters (potential contamination)
other rhodopsins (likely contamination)
Habitat
freshwater
marine
marine invertebrates
salt lake
soil
terrestrial animals

Figure S2. *De novo* multigene species phylogeny of the order Opitutales *sensu* GTDB. Names are provided for cultured isolates. GTDB family labels are indicated in bold. Genera with rhodopsin-coding representatives are indicated in color. The tree is rooted at family UBA9783 as in the GTDB backbone tree. Nodes marked with squares have fast bootstrap support values ≥0.95.

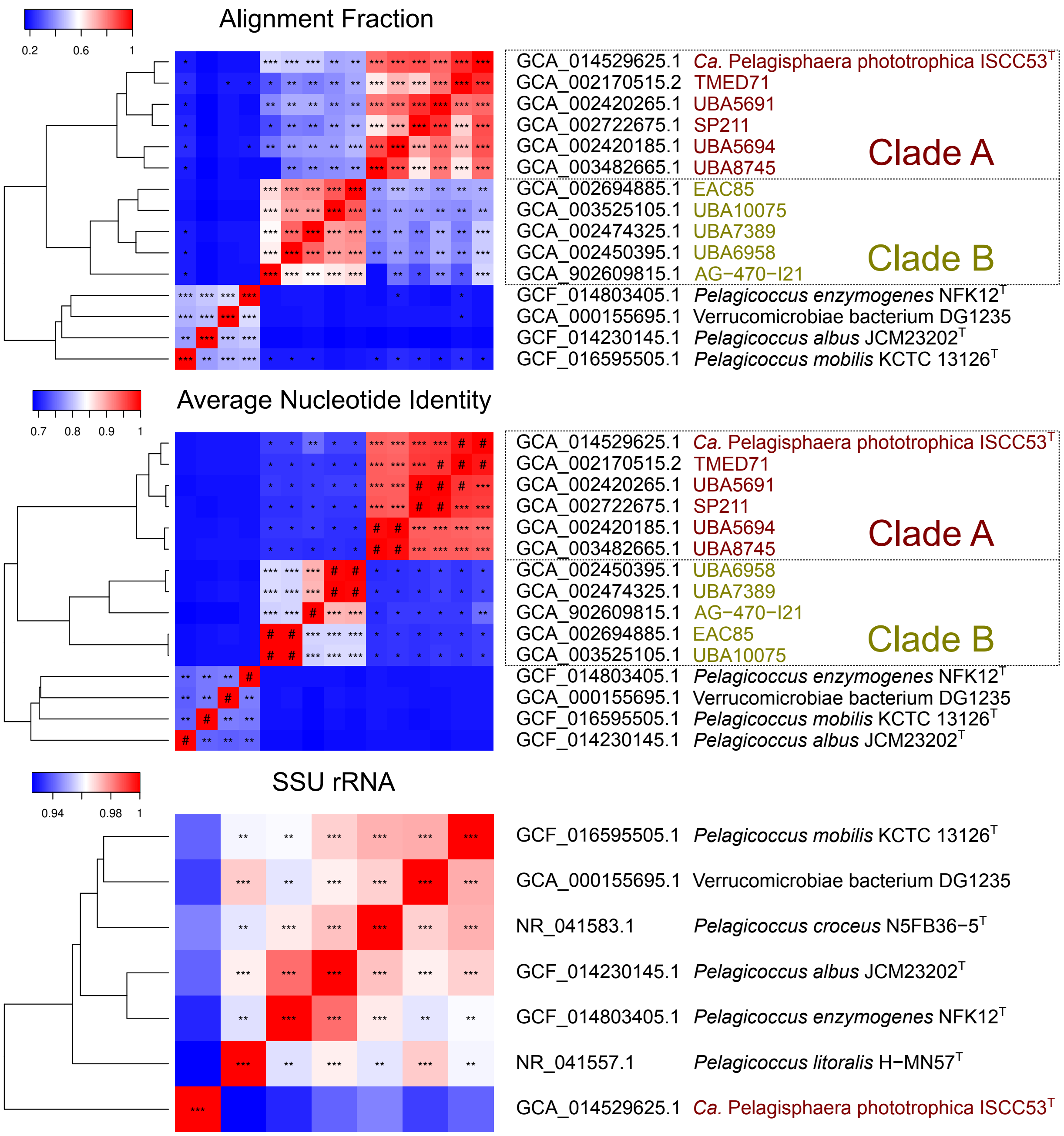

Figure S3. Pairwise comparisons of the key measures used for delineation of *Candidatus* Pelagisphaera: alignment fraction, whole-genome based Average Nucleotide Identity (gANI), 16S rDNA nucleotide identity. The two lineages of *Candidatus* Pelagisphaera are indicated as clades A and B. Symbols indicate values exceeding predefined thresholds known from literature for alignment fraction: "***" >44.4%, "**" >34.5% and "*" >20.6% genus thresholds (25% quartile,

median and 75% quartile, respectively) [8], gANI: "#" >96.5% species threshold [5], "***" >76.56%, "**" >73.11% and "*" >70.85% genus thresholds (25% quartile, median and 75% quartile, respectively) [8], SSU rRNA: "***" >96.4% median genus threshold, "**" >94.5% minimum genus threshold [9].

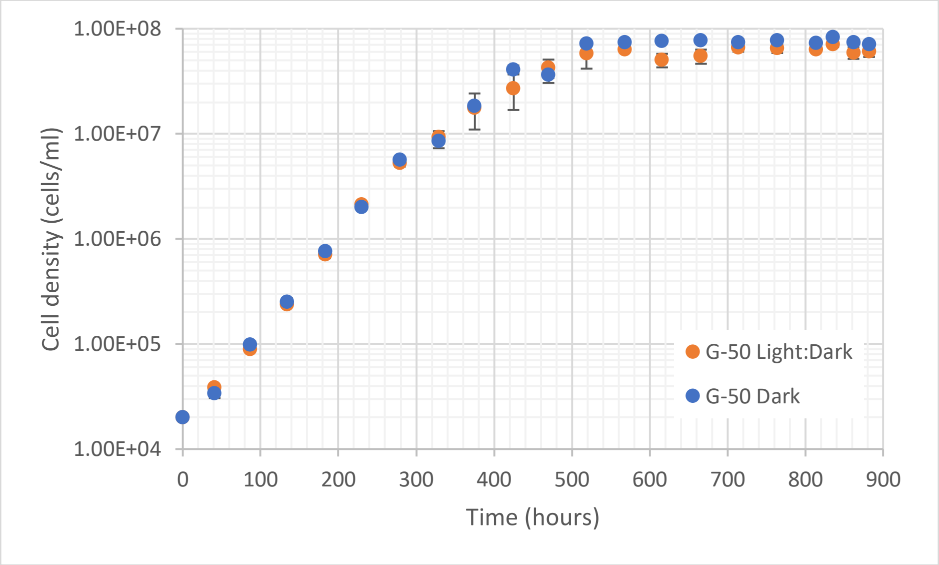

Figure S4: Growth curves of *Ca.* P. phototrophica under light:dark (12:12 h) cycles *versus* continuous dark conditions when grown in ASM-1 medium supplemented with 50 µM glucose. Standard deviations denote biological replicates and are calculated from flow cytometry measurements taken from triplicate cultures.

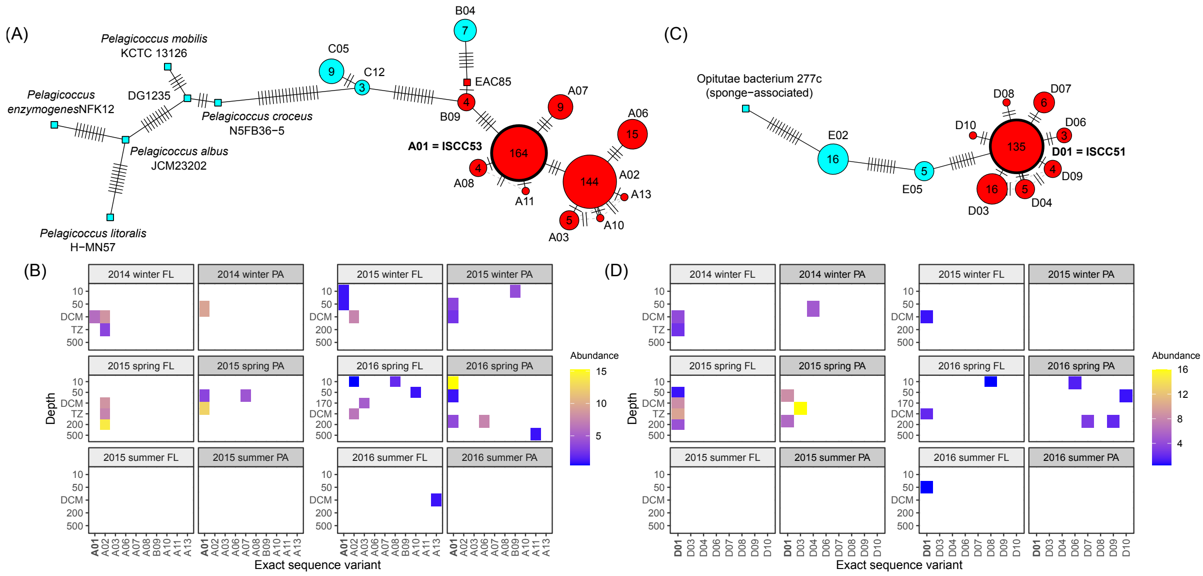

Figure S5: V4-V5 exact sequence variants (ESVs) closely related to the verrucomicrobial strains ISCC53^{T} and ISCC51 from the 16S rRNA gene amplicon data from the Eastern Mediterranean [2]. (A) haplotype network of the ESVs related to *Ca*. Pelagisphaera phototrophica ISCC53^{T} together with 16S rRNA gene sequences from *Pelagicoccus* and (B) their abundance (fragments per 10,000 fragments) at station N1200 during six cruises in three seasons in the free-living (FL) and particle-attached (PA) fractions at different sampling depths. (C) haplotype network and (D) distributions of the ESVs related to strain ISCC51 in the same dataset. Colors in the haplotype networks (A and C) indicate sequence similarity to the V4-V5 fragment of the target isolates, with cyan indicating ≥95% identity and red ≥98%. ESV correspondence to the original numeration in [2] is as follows: A01: ESV00946, 04611; A02: 01085, 02576; A03: 07372; B04: 09302; C05: 09990; A06: 10118; A07: 10317; A08: 11219; B09: 14153; A10: 16470; A11: 16840; C12: 18787, 19339; A13: 21662; D01: 01355, 01712; E02: 04030, 18213; D03: 07655; D04: 09683; E05: 10848; D06: 12177; D07: 13802; D08: 17616; D09: 18245; D10: 20691.

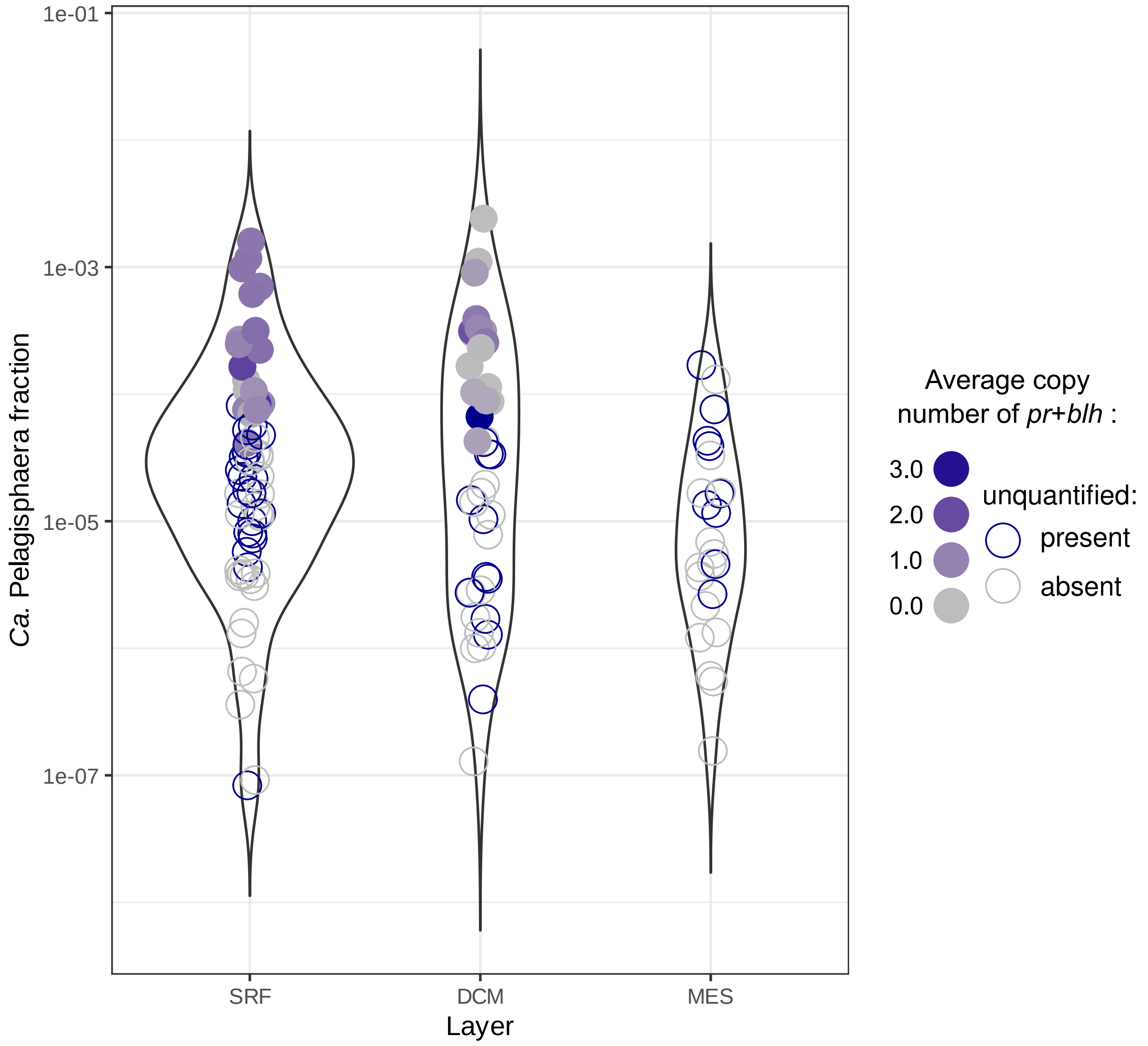

Figure S6: Vertical distribution of *Ca*. P. phototrophica based on the Tara Oceans data (OM-RGC v.2). Each one of the samples is ordinated by one of three layers (SRF - surface, DCM - deep chlorophyll maximum, MES - mesopelagic zone) and the fraction of *Ca.* Pelagisphaera marker genes out of total microbial community. The color reflects the estimated average copy number of the PR gene cluster (averaged over *pr* and *blh*). For stations at which the coverage for the marker genes was low only mere presence and absence of the PR gene cluster coverage is indicated.

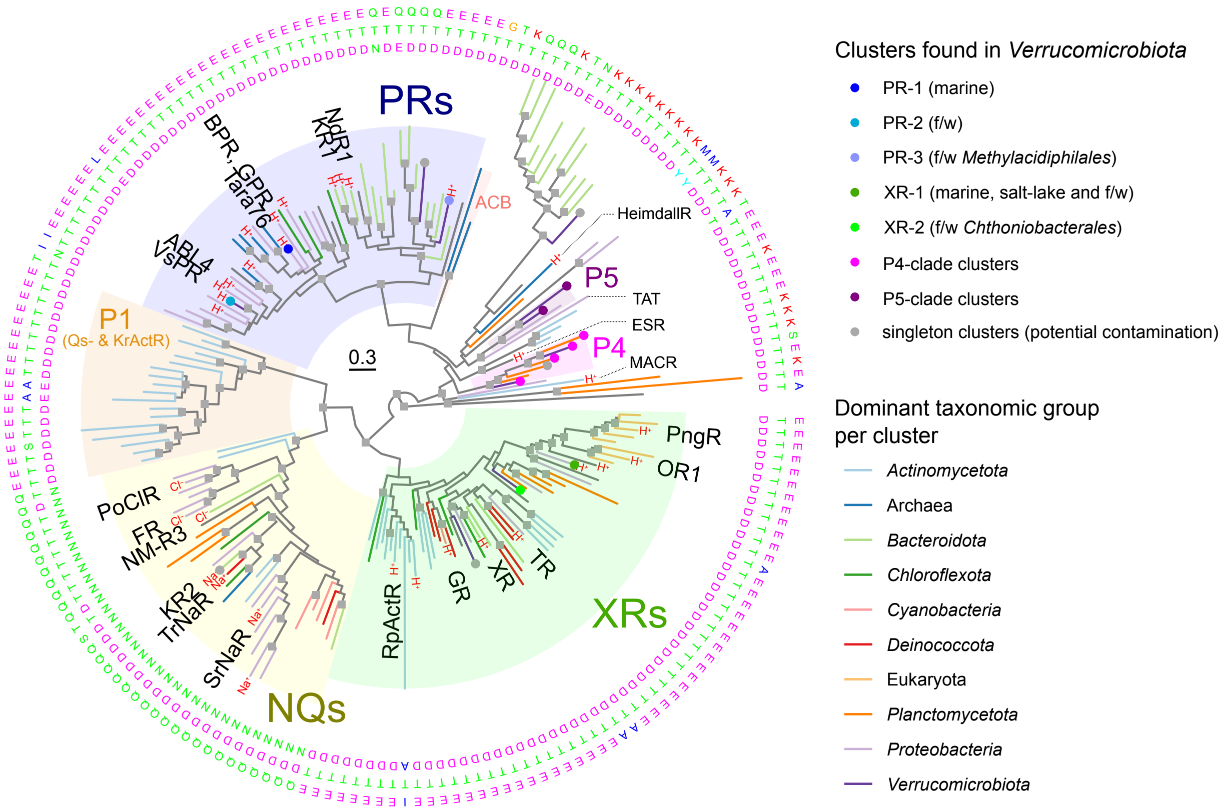

Figure S7: Phylogeny of rhodopsin subfamilies from the PR/XR/NQ superclade. Each tip represents a 60%-identity cluster and is colored by the dominant taxonomic group. Clusters containing characterized rhodopsin pumps are indicated. Some of the named rhodopsins are labeled as well. The outer circle provides TM3 motifs (bacteriorhodopsin positions D85, T89 and D96). Abbreviations of named clades, major: NQs – NQ chloride and sodium pumps, PRs – proteorhodopsins, XRs – xanthorhodopsins, minor: ESR – *Exiguobacterium sibiricum* rhodopsin, HeimdallR – Heimdallarchaeia rhodopsin, MACR – marine actinobacterial clade rhodopsins, P4 – clade P4 (uncharacterized), P5 – clade P5 (uncharacterized), TAT – TAT rhodopsins. The tree is outgroup-rooted, the outgroups are not shown.

References

1. Rappe MS, Connon SA, Vergin KL, Giovannoni SJ. Cultivation of the ubiquitous SAR11 marine bacterioplankton clade. Nature. 2002;418(6898):630-633.
2. Roth Rosenberg D, Haber M, Goldford J, Lalzar M, Aharonovich D, Al-Ashhab A, et al. Particle-associated and free-living bacterial communities in an oligotrophic sea are affected by different environmental factors. Environ Microbiol. 2021;23(8):4295-4308.
3. Camacho C, Coulouris G, Avagyan V, Ma N, Papadopoulos J, Bealer K, et al. BLAST+: architecture and applications. BMC Bioinform. 2009;10:421.
4. Paradis E: pegas: an R package for population genetics with an integrated-modular approach. Bioinformatics. 2010;26(3):419-420.
5. Varghese NJ, Mukherjee S, Ivanova N, Konstantinidis KT, Mavrommatis K, Kyrpides NC, et al. Microbial species delineation using whole genome sequences. Nucleic Acids Res. 2015;43(14):6761-6771.
6. Beja O, Lanyi JK. Nature's toolkit for microbial rhodopsin ion pumps. Proc Natl Acad Sci U S A. 2014;111(18):6538-6539.
7. Dioumaev AK, Petrovskaya LE, Wang JM, Balashov SP, Dolgikh DA, Kirpichnikov MP, et al. Photocycle of *Exiguobacterium sibiricum* rhodopsin characterized by low-temperature trapping in the IR and time-resolved studies in the visible. J Phys Chem B. 2013;117(24):7235-7253.
8. Barco RA, Garrity GM, Scott JJ, Amend JP, Nealson KH, Emerson D. A Genus Definition for Bacteria and Archaea Based on a Standard Genome Relatedness Index. mBio. 2020;11(1).
9. Yarza P, Yilmaz P, Pruesse E, Glockner FO, Ludwig W, Schleifer KH, et al. Uniting the classification of cultured and uncultured bacteria and archaea using 16S rRNA gene sequences. Nat Rev Microbiol. 2014;12(9):635-645.
